# Computational prediction of the interaction network between long non-coding RNAs and microRNAs in maize based on the transcriptome of the fuzzy tassel mutant line

**DOI:** 10.18699/vjgb-25-136

**Published:** 2025-12

**Authors:** J. Yan, A.Yu. Pronozin, D.A. Afonnikov

**Affiliations:** Novosibirsk State University, Novosibirsk, Russia; Institute of Cytology and Genetics of the Siberian Branch of the Russian Academy of Sciences, Novosibirsk, Russia Kurchatov Genomic Center of ICG SB RAS, Novosibirsk, Russia; Novosibirsk State University, Novosibirsk, Russia Novosibirsk State University, Novosibirsk, Russia Kurchatov Genomic Center of ICG SB RAS, Novosibirsk, Russia

**Keywords:** lncRNA, miRNA, gene regulation, maize, fuzzy tassel (fzt), DCL1, bioinformatics, RNA interaction, competing endogenous RNA (ceRNA, днРНК, миРНК, регуляция генов, кукуруза, мутация fzt, DCL1, биоинформатика, взаимодействие РНК, конкурирующие эндогенные РНК

## Abstract

Long non-coding RNAs (lncRNAs) play an important role in the regulation of gene expression, including interactions with microRNAs (miRNAs), acting as molecular “sponges”. Bioinformatics methods are generally used to predict such interactions. To refine computational predictions, additional evidence based on the co-expression of miRNAs and lncRNAs can be incorporated. In the present study, we investigated potential interactions between lncRNAs and miRNAs in the maize mutant line fuzzy tassel (fzt), which is characterized by reduced expression of certain miRNAs due to a mutation in the Dicer-like1 (DCL1) gene in shoot and tassel tissues. Transcriptome assembly was performed based on RNA-seq data from maize shoot and tassel tissues of control and mutant lines, with data obtained from the NCBI SRA archive. In the shoot, 10 lncRNAs with significantly altered expression levels between control and mutant groups were identified, 9 of which were upregulated in the mutant plants. In the tassel, 34 differentially expressed lncRNAs were identified, with 20 showing increased expression in the mutant line. For lncRNAs with increased expression and miRNAs with decreased expression in the mutant line, potential interactions were predicted using the machine learning algorithm PmliPred. The IntaRNA program was used to confirm possible complementary binding for the identified miRNA–lncRNA pairs, which enabled the construction of competing endogenous RNA (ceRNA) networks. Structural analysis of these networks revealed that certain lncRNAs are capable of binding multiple miRNAs simultaneously, supporting their regulatory role as “sponges” for miRNAs. The results obtained deepen our understanding of post-transcriptional regulation in maize and open new perspectives for breeding strategies aimed at improving stress tolerance and crop productivity.

## Abstract (ru)

Длинные некодирующие РНК (днРНК) играют важную роль в регуляции экспрессии генов, включая взаимодействия с микроРНК (миРНК), выполняя функцию молекулярных «губок». Для предсказания таких взаимодействий, как правило, применяются методы биоинформатики. Для уточнения предсказаний компьютерных программ можно использовать дополнительные данные на основе коэкспрессии миРНК и днРНК. В настоящей работе исследуются потенциальные взаимодействия между днРНК и миРНК у мутантной линии кукурузы fuzzy tassel (fzt), характеризующейся сниженной экспрессией некоторых миРНК вследствие мутации в гене Dicer-like1 (DCL1) в тканях побега и соцветия. Проведена сборка транскриптомов на основе данных RNA-seq побега и соцветия кукурузы контрольной и мутантной линий. Данные были взяты из архива SRA NCBI. Для побега было идентифицировано десять днРНК, достоверно изменяющих свой уровень экспрессии между контрольной и мутантной группами, девять из них повышают экспрессию у мутантных растений. Для соцветия идентифицировано 34 дифференциально экспрессирующихся днРНК (20 с повышенным уровнем экспрессии у мутантных линий). Для днРНК с повышенным уровнем собственной экспрессии и миРНК с пониженным уровнем экспрессии в мутантных линиях были предсказаны потенциальные взаимодействия с помощью алгоритма машинного обучения PmliPred. С использованием программы IntaRNA подтверждена возможность комплементарного связывания для выявленных пар миРНК–днРНК, что позволило построить конкурирующие эндогенные РНК-сети. Анализ структуры этих сетей показал, что отдельные днРНК способны связывать несколько миРНК одновременно, подтверждая их регуляторную функцию в качестве «губок» для миРНК. Полученные результаты углубляют понимание посттранскрипционной регуляции у кукурузы и открывают перспективы для селекционных разработок, направленных на повышение стрессоустойчивости и продуктивности растений

## Introduction

In recent years, the rapid development of next-generation high-throughput sequencing technologies has enabled the identification of tens of thousands of non-protein-coding transcripts (Sheng et al., 2023). Initially, these sequences were considered transcriptional noise. However, subsequent studies have revealed that approximately 75 % of cellular transcripts lack protein-coding potential, yet they actively participate in the regulation of gene expression (Wang L., Wang J.W., 2015). Non-coding RNAs (ncRNAs) are generally classified into housekeeping and regulatory types. Regulatory ncRNAs can be further divided into small and long non-coding RNAs based on their transcript length (Li R. et al., 2016). To date, the biological functions of small ncRNAs, particularly microRNAs (miRNAs), have been extensively studied; they are capable of repressing mRNA expression at both transcriptional and post-transcriptional levels. In contrast, the functions of long non-coding RNAs (lncRNAs) remain poorly understood, especially in plants

Recent studies have revealed that lncRNAs and miRNAs engage in complex interactions that play crucial roles in numerous biological processes. Several mechanisms underlying these interactions have been identified (Pronozin, Afonnikov, 2025). For example, lncRNAs can function as molecular “sponges”, binding complementarily to miRNAs and thereby preventing their interaction with target mRNAs. Such interactions contribute to the regulation of plant growth, development, tissue differentiation, and stress responses. However, due to the limited scale of experimental studies, bioinformatic approaches are increasingly needed to identify these interactions (Sheng et al., 2023).

To date, the PmliPred method has been developed to identify interactions between lncRNAs and miRNAs (Kang et al., 2020). This method is based on deep learning for predicting molecular interactions. Information on potential miRNA– lncRNA interactions can be valuable for modeling regulatory networks involved in gene expression. Furthermore, the obtained results can serve as a basis for subsequent functional experiments and may have practical applications in breeding programs. It should also be noted that potential miRNA– lncRNA interactions can be inferred from co-expression analyses (He et al., 2020).

The present study aims to identify interactions between lncRNAs and miRNAs in maize using bioinformatic approaches, taking into account co-expression data of miRNAs and lncRNAs. The fuzzy tassel ( fzt) mutant line of maize, which exhibits disrupted miRNA biogenesis due to a mutation in the Dicer-like1 (DCL1) gene, a key player in the processing of miRNA precursors, was used as a model for this study (Thompson et al., 2014). Impaired DCL1 function leads to reduced levels of several mature miRNAs, which in turn causes an imbalance in regulatory interactions and, consequently, in the expression of miRNAs and their target mRNAs (Thompson et al., 2014). We hypothesize that the decreased concentration of miRNAs may reduce the formation of duplexes with lncRNAs that act as molecular “sponges”. In this scenario, the degradation rate of lncRNA “sponges” would decrease, leading to an increase in their abundance. Thus, similar to mRNAs exhibiting elevated expression in the fzt maize line (Thompson et al., 2014), lncRNAs with increased levels in this line may serve as targets of these miRNAs. The results obtained from this study are expected to enhance our understanding of post-transcriptional regulation in plants and may inform the development of novel breeding strategies aimed at improving stress tolerance and crop productivity (Zhang L. et al., 2009; Sun Q. et al., 2013).

## Materials and methods

Transcriptome data. In this study, RNA-seq data were obtained from the open NCBI Sequence Read Archive (SRA) database (accession numbers GSM1277448–GSM1277461, see the Table) (Thompson et al., 2014). The samples were divided into two groups: control and mutant. The mutant lines contained a deletion in the Dicer-like1 (DCL1) gene, which plays a key role in the processing of miRNA precursors. Gene expression was assessed separately for whole seedling and tassel tissues, including both long RNAs and miRNAs.

As shown previously (Thompson et al., 2014), expression of 22 miRNAs was significantly reduced in the seedling (miR398b-5p, miR408a-b-3p, miR408b-5p, miR394a-b-5p, miR167c-3p*, miR156a-3p*, miR167b-3p*, miR319b,d-5p*, miR169i-k-5p, miR167a-d-5p, miR168b-3p*, miR168a-3p*, miR156d-f-g-3p*, miR398a-b-3p, miR528a-b-3p, miR156e- 3p*, miR397a-b-5p, miR159a-5p, miR2118b, miR399e,i-j-3p, miR160a-e,g-5p, miR398a-5p*) and 14 miRNAs in the tassel (miR167d-3p*, miR167a-d-5p, miR172e, miR408a-b-3p, miR398b-5p*, miR394a-b-5p, miR167c-3p*, miR398a-b-3p, miR319a-d-3p, miR159a-b,f,j-k-3p, miR528a-b-5p, miR160ae, g-5p, miR166j-k,n-3p, miR159a-5p*)

The reference genome of maize (Zea mays) version 5 (Zm-B73-REFERENCE-NAM-5.0) was used in this study, downloaded along with its annotation from the Ensembl Plants database (Bolser et al., 2016)MiRNA sequences were obtained from miRBase version 22.1 (https://www.mirbase.org/).

Bioinformatics analysis. This study consisted of two main blocks of bioinformatics analysis: transcriptome assembly followed by the differential expression analysis of lncRNAs; prediction of miRNA–lncRNA interactions using deep learning– based approaches. A detailed description of each analytical step is provided below

**Fig. 1.**
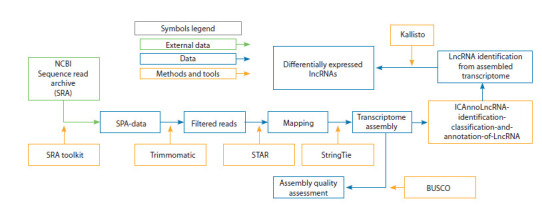
Workflow of the bioinformatics pipeline for maize transcriptome assembly Green rectangles represent the description of external data sources; blue rectangles indicate library data and intermediate results, and orange rectangles denote software tools used in the analysis

Transcriptome assembly and analysis of maize. Transcriptome assembly (Fig. 1) included the following steps: data preprocessing, transcriptome assembly, identification and annotation of lncRNAs, and quantification of transcript expression levels.

Read filtering was performed using Trimmomatic (Bolger et al., 2014) with the following parameters: removal of adapter sequences, elimination of short reads shorter than 36 nucleotides, and quality-based trimming of low-quality reads. After preprocessing, the filtered reads were aligned to the Z. mays reference genome using STAR (Dobin et al., 2013). Based on the alignment results, transcriptome assembly was conducted using StringTie (Pertea et al., 2015). The completeness and quality of the assembled transcriptome were evaluated with BUSCO (Simão et al., 2015). Identification and annotation of lncRNAs were performed using ICAnnoLncRNA (Pronozin, Afonnikov et al., 2023). Expression levels of identified lncRNAs and other transcripts were quantified using Kallisto (Bray et al., 2016).

Differential expression analysis of lncRNAs in maize. Differential expression analysis of lncRNAs was performed separately for shoot and inflorescence tissues by comparing wild-type (control) and mutant ( fzt) maize lines. Statistical analysis was conducted using the DESeq2 and edgeR packages (Robinson et al., 2010; Love et al., 2014). Transcripts were considered significantly differentially expressed at a p-value < 0.05, adjusted for multiple testing

For the differentially expressed lncRNAs, heatmaps of normalized expression values were generated to visualize expression patterns across biological replicates and to confirm the consistency of expression changes between the control and mutant groups

**Fig. 2.**
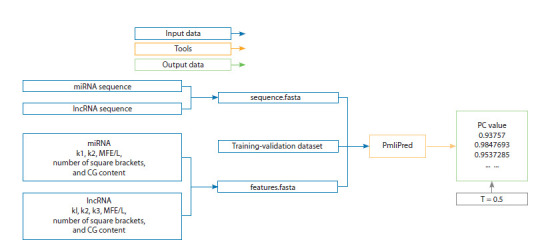
Workflow of predicting interactions between lncRNAs and miRNAs using the PmliPred model Blue rectangles represent the input data, green rectangles indicate the output results, and orange rectangles denote the software tools used in the analysis. The threshold value of confidence probability (T = 0.5) is shown.

Analysis of interactions between miRNAs and lncRNAs. Interactions between miRNA and lncRNA molecules were predicted using the PmliPred method (Kang et al., 2020), which involves several consecutive analytical stages (Fig. 2). At the first stage, input data were prepared, including nucleotide sequences of miRNAs and lncRNAs that exhibited downregulated miRNA expression and upregulated lncRNA expression in mutant plants compared with the control. The input to the program also included quantitative sequence features extracted by the built-in algorithms of the model, as well as the training dataset provided with the software package (Kang et al., 2020). For miRNAs, the following features were used: k-mer frequencies (k = 1, k = 2), minimum free energy normalized by length (MFE/L), number of paired nucleotides in the secondary structure, and GC content ratio. For lncRNAs, an additional feature representing k-mer frequencies (k = 3) was extracted

The processed data were analyzed using the PmliPred program to estimate the interaction probability between miRNA– lncRNA pairs (output parameter PC, confidence probability). A miRNA–lncRNA pair was considered to have a potential interaction when the PC value was ≥ 0.5. The results were presented in a table containing probability scores, which reflected the predicted strength of interaction between miRNA and lncRNA molecules

**Table 1.**
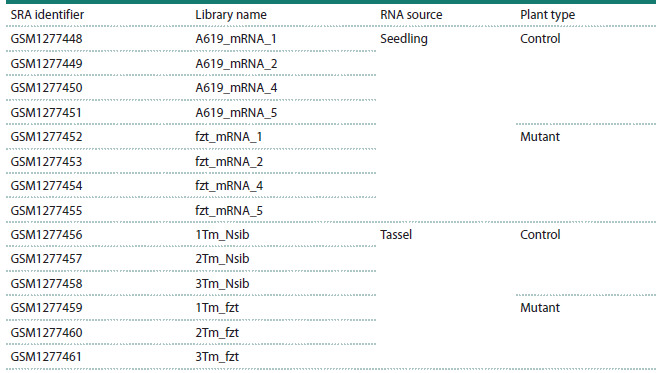
RNA-seq libraries of maize (Zea mays) obtained from seedling and tassel tissues of control plants and the fuzzy tassel (fzt) mutant line

Analysis and visualization of interactions between miRNAs and lncRNAs. The obtained miRNA–lncRNA pairs were divided into two groups based on their interaction parameters: lncRNAs with increased expression levels in the mutant line (test group) and lncRNAs with decreased expression levels (control group). Both groups of lncRNAs were compared with all miRNAs showing reduced expression levels, as reported by Thompson et al. (2014) (see section “Transcriptomic data”). As a threshold for selecting potential interactions in the test group, the maximum value of the PC parameter calculated by the PmliPred program for the control group was used. If for a given miRNA–lncRNA pair from the test group, the PC parameter exceeded any of the PC values from the control group, such miRNA–lncRNA pairs were considered to interact.

The sequences of the selected miRNAs and lncRNAs were uploaded into the IntaRNA program (Mann et al., 2017) for the identification and visualization of base-pairing interactions. Among all predicted interactions, only those pairs were retained, in which the number of unpaired nucleotides within the interaction region of the two molecules was fewer than 4, and the length of the interaction region exceeded 16 nucleotides

Such interactions between lncRNAs and miRNAs have important biological significance. lncRNAs can function as competing endogenous RNAs (ceRNAs), or “sponges”, by binding to miRNAs and thereby preventing them from interacting with their mRNA targets. This mechanism contributes to the regulation of gene expression involved in plant growth, development, and stress responses (Pronozin, Afonnikov, 2025).

## Results

**Transcriptome assembly**

As a result of the transcriptomic analysis of Z. mays, covering both seedling and tassel stages for control and mutant ( fzt) lines, high-quality raw data were obtained. The average percentage of uniquely mapped reads during alignment using STAR (Dobin et al., 2013) was 84.73 %, while only 3.10 % of reads remained unmapped. For the aligned reads, the average mismatch rate per nucleotide was 0.76 %, indicating high sequencing accuracy and the reliability of the data for subsequent analyses.

The transcriptome assemblies generated using StringTie (Pertea et al., 2015) were evaluated with the BUSCO tool (Simão et al., 2015). In all 14 libraries, the proportion of complete BUSCO groups exceeded 95 %, reaching a maximum of 98.8 % (252 out of 255 expected orthologs detected in library SRR1041561). These metrics indicate the completeness and high quality of the obtained assemblies, confirming their suitability for subsequent expression analysis and the identification of noncoding RNAs.

**Differential expression of lncRNAs between control and mutant Z. mays samples**

In seedling tissue, 10 lncRNAs were identified as significantly differentially expressed between the control and mutant groups (Table S1)1. Among these, nine lncRNAs showed increased expression in the mutants, suggesting that they may serve as targets for miRNAs and participate in post-transcriptional regulatory mechanisms. These transcripts were subsequently considered as candidate miRNA targets in further analyses

Supplementary Materials are available in the online version of the paper: https://vavilovj-icg.ru/download/pict-2025-29/appx51.pdf


**Fig. 3.**
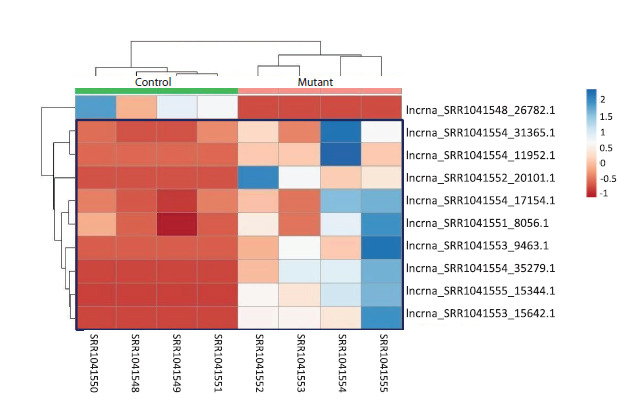
Heatmap of differentially expressed lncRNAs in seedling tissue Here and in Fig. 4: the color scale on the right represents normalized expression levels, with blue indicating high expression, and red indicating low expression. Cells corresponding to lncRNAs with increased expression in the mutant line are highlighted with a blue frame.

The heatmap (Fig. 3) illustrates systematic differences in the expression of these lncRNAs across the analyzed transcriptomic libraries. For 9 out of the 10 lncRNAs, expression levels were higher in the mutant plants compared with the control.

In tassel tissue, the number of differentially expressed lncRNAs was considerably higher, with a total of 34 lncRNAs identified (Table S2). Among these, 20 lncRNAs exhibited increased expression in the mutant line. Notably, pronounced differences in transcription levels were observed for several lncRNAs that displayed strong tissue-specific expression patterns unique to the tassel.

**Fig. 4.**
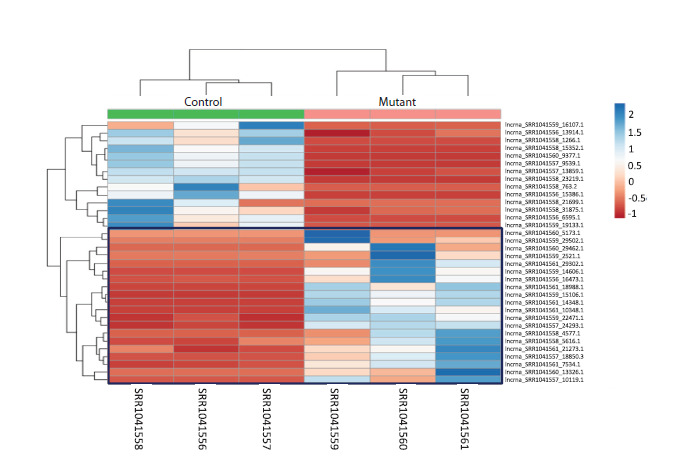
Heatmap of differentially expressed lncRNAs in tassel tissue

The heatmap of lncRNA expression in tassel tissue (Fig. 4) also illustrates systematic differences across the analyzed transcriptomic libraries. lncRNAs with decreased and increased expression levels in the mutant plants formed two clearly distinct clusters.

Overall, the identified lncRNAs represent a prioritized set for subsequent analysis of interactions with miRNAs and for further functional annotation.

**Assessment of the accuracy of miRNA–lncRNA interaction predictions**

**Fig. 5.**
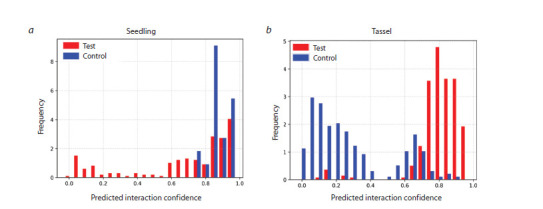
Distribution of predicted miRNA–lncRNA interaction scores in seedlings and tassel tissues a – seedlings: interaction scores for the test group (lncRNAs with increased expression) are shown in red, and for the control group (lncRNAs with decreased expression), in blue; b – tassel: similarly, red represents the test group, and blue, the control group (lncRNAs with decreased expression). The X-axis represents the predicted interaction confidence (PC) calculated by PmliPred, and the Y-axis indicates the number of miRNA–lncRNA pairs analyzed.

The evaluation of the model’s ability to distinguish lncRNAs from the test group (with increased expression in mutants) from those in the control group (with decreased expression) is presented in Fig 5.

In seedling tissue, the interaction scores for the test lncRNAs (with increased expression in mutants) were shifted above 0.5, suggesting a potential ability of these transcripts to bind miRNAs. However, it should be noted that the control group contained only a single lncRNA with a high predicted score. Because the control in this experiment consisted of only one lncRNA (with decreased expression in mutants), it was difficult to accurately assess the precision and discriminatory power of the PmliPred model

In tassel tissue, the differences between the groups were even more pronounced: interaction scores for the test lncRNAs were predominantly above 0.5, whereas the control lncRNAs displayed a distribution shifted below 0.5. This behavior of the model indicates its ability to effectively distinguish biological classes based on the predicted miRNA–lncRNA interaction parameters.

Thus, the PmliPred model demonstrated high discriminatory power and can be used for the preliminary selection of lncRNAs potentially involved in interactions with miRNAs

**miRNA–lncRNA interaction networks in maize tissues**

**Fig. 6.**
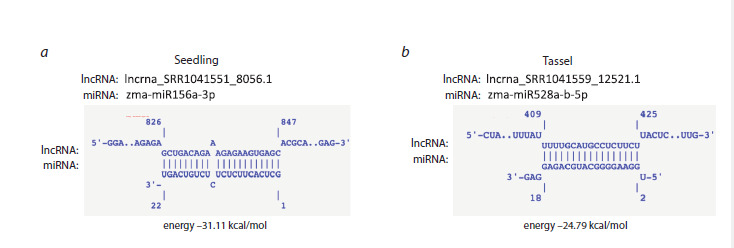
Examples of miRNA–lncRNA interactions predicted using IntaRNA a – seedlings: zma-miR156a-3p and lncRNA_mapped_SRR1041551_8056.1; b – tassel: zma-miR528a-b-5p and lncRNA_mapped_SRR1041559_ 12521.1. Regions of base pairing and the interaction structures are shown, calculated based on minimum free energy (kcal/mol).

The results obtained using the miRNA–lncRNA interaction prediction tool IntaRNA are shown in Fig. 6. For example, two RNA pairs clearly formed stable and extensive regions of complementary binding. In total, 13 reliable miRNA–lncRNA pairs were identified in seedling tissue, and 14 pairs, in tassel tissue. These data confirm that the selected lncRNAs not only exhibit increased expression in the mutants but also possess a high potential for specific interactions with miRNAs, the expression of which is reduced in the mutants. This makes them justified candidates as miRNA targets.

**Fig. 7.**
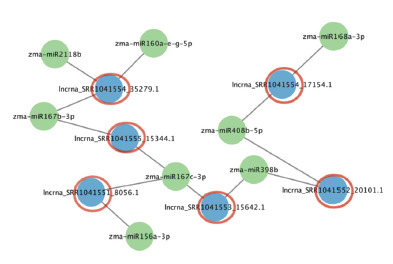
miRNA–lncRNA interaction networks in maize seedling tissue Here and in Fig. 8: green circular nodes represent miRNAs, blue circular nodes represent lncRNAs, and red circles indicate lncRNAs that are potentially functioning as “sponges”.

**Fig. 8.**
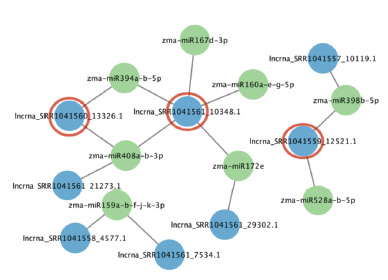
miRNA–lncRNA interaction networks in maize tassel tissue

Visualization of the identified interactions using Cytoscape (Figs. 7 and 8) revealed that some lncRNAs are potentially capable of binding multiple miRNAs simultaneously. For example, lncrna_SRR1041561_10348.1 (in tassel) interacts with five different miRNAs: zma-miR160a-e-g-5p, zmamiR167d- 3p, zma-miR394a-b-5p, zma-miR408a-b-3p, and zma-miR172e, suggesting its potential role as a “sponge” within a ceRNA mechanism. Another example is lncrna_ SRR1041554_35279.1 in seedling tissue, which interacts with zma-miR2118b, zma-miR160a-e-g-5p, and zma-miR167b-3p.

## Discussion

The analysis revealed that 9 out of 14 identified lncRNAs are potentially capable of interacting with multiple miRNAs simultaneously, suggesting their possible role as competing endogenous RNAs (ceRNAs), molecular “sponges” that bind miRNAs and prevent their interaction with target mRNAs. Through this mechanism, lncRNAs can indirectly regulate the expression of various genes involved in key biological processes.

Among the predicted miRNA partners are well-characterized regulators of plant growth, development, and stress responses (Jones-Rhoades et al., 2006; Sunkar et al., 2012): • miR156 regulates the transition from the juvenile to the adult phase, flowering, leaf morphogenesis, and branching by suppressing SPL genes (Preston et al., 2013; Wang H., Wang H., 2015); • miR167 and miR160 regulate the auxin signaling pathway by suppressing ARF genes, thereby influencing root formation, leaf, flower, and seed development, as well as somatic embryogenesis (Caruana et al., 2020; Barrera-Rojas et al., 2021; Wang Y. et al., 2020); • miR168 participates in maintaining the stable level of the AGO1 protein, a central component of the RNA interference (RNAi) machinery, thereby regulating the entire miRNA pathway (Martínez de Alba et al., 2011; Li W. et al., 2012); miR172 regulates the onset of flowering and organogenesis by repressing AP2-type transcription factor genes (Ripoll et al., 2015; Zhang B. et al., 2015); • miR2118 activates the biogenesis of phased small interfering RNAs (phasiRNAs), playing a critical role in plant immunity and anther development (Canto-Pastor et al., 2019; Jiang P. et al., 2020); • miR398 and miR408 provide antioxidant protection by regulating the levels of superoxide dismutases and metalbinding proteins, and they also respond to a wide range of abiotic stresses (Jiang A. et al., 2021; Zou et al., 2021; Gao et al., 2022); • miR394 influences leaf morphogenesis, fruit development, and meristem activity (Song et al., 2015; Sun P. et al., 2017); • miR528 is involved in redox homeostasis, resistance to viral infections, salt stress response, and regulation of lignification (Wu et al., 2017; Sun Q. et al., 2018).

Functional annotation of the interacting miRNAs indicates that most of them are involved not only in the development of plant morphological structures, but also in the complex regulatory networks controlling responses to biotic and abiotic stresses.

Moreover, the identified ceRNA networks confirm that post- transcriptional regulation in plants is mediated through finely coordinated interactions between non-coding and coding RNAs. The presence of lncRNAs capable of binding multiple regulatory miRNAs suggests the existence of potential hubs of regulatory cross-talk within RNA networks, which represents a particularly promising target for functional validation.

The obtained results emphasize the importance of a systems- level approach to transcriptomic data analysis, as such strategies enable the identification of hidden layers of gene regulation and promising molecular targets. Furthermore, these findings may serve as a theoretical foundation for the development of new agronomically valuable maize varieties with enhanced stress tolerance and improved adaptive traits.

## Conclusion

In this study, a comprehensive analysis of Z. mays transcriptomic data was conducted to identify potential interactions between miRNAs and lncRNAs. Based on the results of differential expression analysis comparing control and mutant samples, lncRNAs and miRNAs with potential interactions were identified.

The PmliPred model, based on machine learning approaches, was applied to predict potential miRNA–lncRNA pairs. Subsequent structural analysis using IntaRNA confirmed the presence of stable complementary binding sites between the selected molecules, indicating high reliability of the predicted interactions.

Based on the selected interaction pairs, competing endogenous RNA (ceRNA) networks were constructed, demonstrating that individual lncRNAs are capable of binding multiple miRNAs simultaneously. This supports the hypothesis that they participate in post-transcriptional regulatory mechanisms as miRNA “sponges”, capable of modulating the activity of regulatory molecules and influencing the expression of target genes

Additionally, key interactions were visualized using Cytoscape, allowing a clear representation of the structure and potential functional significance of the identified regulatory connections. The results confirm the role of lncRNAs as important components of plant regulatory networks and provide a foundation for further functional studies.

## Conflict of interest

The authors declare no conflict of interest.
